# Granulome centro-facial révélant une syphilis tertiaire

**DOI:** 10.11604/pamj.2013.15.82.3011

**Published:** 2013-06-30

**Authors:** Karima Chakir, Hakima Benchikhi

**Affiliations:** 1 Service de Dermatologie et vénérologie CHU Ibn Rochd Casablanca, Maroc

**Keywords:** Granulome, centro-facial, syphilis tertiaire, granuloma, centro-facial, tertiary syphilis

## Image en médecine

Les granulomes ou processus centro-faciaux sont caractérisés par une double définition : Clinique par la présence de lésions ulcéro-nécrotiques extensives siégeant au niveau des voies aériennes supérieures évoluant vers la destruction de la région médio-faciale; et histologiquement par la présence d'un granulome épithélio ‘giganto cellulaire. Les étiologies sont multiples: infectieuses, inflammatoires et tumorales; ce qui pose des difficultés diagnostiques source de retard de prise en charge. Nous rapportons le cas de Mr S.M âgé de 32 ans; qui consulte pour un processus centro-facial évoluant depuis trois ans. Le début était marqué par l'apparition d'un petit nodule de lèvre sup à droite, indolore augmentant progressivement de volume puis extension à toute la région médio-faciale; dans un contexte d'apyrexie et de conservation de l’état général. L'examen dermatologique a objectivé un placard infiltré inflammatoire de couleur rouge cuivrée ou siège plusieurs cicatrices rétractiles d'anciennes ulcérations; une fissure longitudinale de l'arête nasale donnant issu à des sérosités jaunatres; l'examen ORL a objectivé une voix nasonnée; une gêne respiratoire; une limitation de l'ouverture buccale; destruction de la cloison nasale; un rétrécissement des orifices narinaires; des adénopathies sous mentonnières non inflammatoires, Le reste de l'examen somatique était sans anomalie. une biopsie cutanée a montré plusieurs granulomes épithélio-giganto cellulaires sans nécrose caséeuse; et un infiltrat lympho-plasmocytaire; le bilan de tuberculose était négatif; la sérologie syphilitique était très positive;la ponction lombaire était normale; les prélèvements bactériologiques et mycologiques étaient négatifs; les ANCA étaient négatives; la TDM faciale n'a pas montré d'atteinte osseuse sous jacente;on a retenu le diagnostic de syphilis tertiaire cutanée et le patient a été mis sous pénicilline retard avec bonne évolution; et disparition de la gêne respiratoire et de la voix nasonnée et de l'inflammation cutanée.

**Figure 1:**
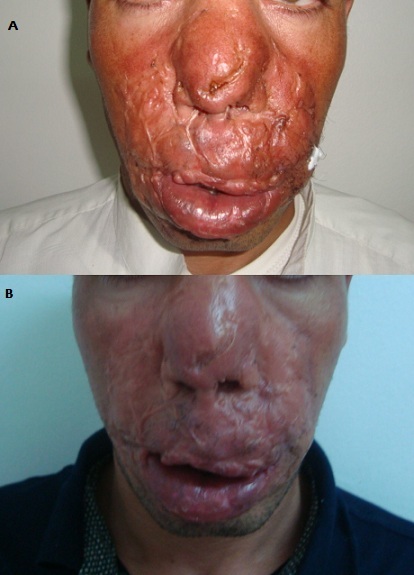
A) Granulome centrofacial:syphilis tertiaire; B) Evolution aprés pénicillinothérapie

